# Association between three-timepoint maternal blood pressure trajectories during pregnancy and low birth weight: a longitudinal study based on NHANES 2005–2006

**DOI:** 10.3389/fped.2025.1604845

**Published:** 2025-06-19

**Authors:** Huiqiang Liu, Yanping Wei, Wen Zhang, Fei Meng, Nina Ding, Xicui Liu

**Affiliations:** Department of Obstetrics and Gynecology, Second Hospital of Shanxi Medical University, Taiyuan, Shanxi, China

**Keywords:** pregnancy blood pressure trajectories, low birth weight (LBW), group-based trajectory modeling (GBTM), directed acyclic graph (DAG), systolic blood pressure (SBP), maternal risk factors

## Abstract

**Background:**

Dynamic changes in maternal blood pressure influence neonatal birth weight however, studies investigating the association between blood pressure trajectories during pregnancy and low birth weight (LBW) remain limited. This study aims to identify maternal blood pressure trajectories based on three time points using group-based trajectory modeling (GBTM) and explore their association with LBW.

**Methods:**

This study was based on the NHANES 2005–2006 database and included 330 pregnant women meeting the eligibility criteria (41 cases in the LBW group and 289 in the control group). GBTM was applied to model three blood pressure measurements [systolic blood pressure (SBP), diastolic blood pressure (DBP), pulse pressure (PP)] taken during pregnancy. Multilevel logistic regression was used to assess the relationship between blood pressure trajectories and LBW. Additionally, stratified analyses were conducted to evaluate the modifying effects of age, body mass index (BMI), and education level, and directed acyclic graph (DAG) were employed for covariate selection.

**Results:**

Three distinct blood pressure trajectory patterns were identified. Logistic regression revealed that, compared with the low blood pressure trajectory, mothers with a high-medium SBP trajectory had a significantly increased risk of delivering an LBW infant [odds ratio [OR] = 4.479, 95% confidence interval [CI]: 2.541–7.895, *P* < 0.001]. Stratified analyses indicated that this association was more pronounced in mothers who were older than 40 years, had a BMI >28, had lower income, did not consume alcohol, and had abnormal cholesterol levels. The DAG analysis further supported the independent effect of blood pressure trajectories on LBW.

**Conclusions:**

Maternal blood pressure trajectories based on three prenatal measurements are closely associated with LBW, particularly among mothers with a high-medium SBP trajectory. This study underscores the importance of monitoring blood pressure fluctuations during pregnancy and suggests that early intervention may help reduce the risk of LBW.

## Introduction

Low birth weight (LBW) refers to a newborn's birth weight of less than 2,500 g (1). It is not only a significant factor influencing neonatal mortality and morbidity, but is also associated with an increased risk of long-term metabolic diseases, cardiovascular diseases, and neurodevelopmental disorders (2–4). Maternal blood pressure changes during pregnancy may affect placental perfusion and fetal growth, thereby having a significant impact on neonatal birth weight (5, 6). Previous studies have shown that hypertensive disorders of pregnancy (HDP) significantly increase the risk of LBW (7). However, even in pregnant women without HDP, the dynamic changes in blood pressure (trajectories) may still have a potential impact on LBW (8, 9). For example, Teng et al. (10) examined the association between systolic blood pressure (SBP) trajectories and a range of pregnancy outcomes, including low birth weight (LBW), and found that variations in SBP patterns were significantly associated with LBW risk. Building upon this line of research, our study focuses specifically on the relationship between SBP trajectories and LBW by applying a data-driven clustering approach to classify SBP change patterns over time, aiming to better capture dynamic maternal blood pressure profiles during pregnancy. Currently, research on the relationship between blood pressure trajectories during pregnancy and LBW is still limited, especially regarding long-term blood pressure patterns in non-HDP populations and their impact on LBW. Therefore, exploring maternal blood pressure trajectories during pregnancy and evaluating their potential effects on LBW is of significant clinical importance. The methodological innovation of this study lies in the first-time application of Group-based trajectory modeling (GBTM) and Directed Acyclic Graphs (DAG) to study maternal blood pressure trajectories during pregnancy and their association with LBW.

GBTM has been widely applied in epidemiology and public health to analyze health parameter patterns over time, allowing researchers to identify different blood pressure trajectory groups and revealing long-term trends in maternal blood pressure (11–13). Although Linear Mixed Effects (LME) models could also analyze longitudinal data, GBT was chosen because it better captures the heterogeneous, non-linear changes in blood pressure over time, whereas LME assumes a single, linear trajectory for the entire population. This method offers a novel perspective for early prediction and intervention to prevent LBW (14). Moreover, this study is the first to use DAG methodology for covariate selection in this context, with the goal of reducing, confounding, optimizing model specification, and informing suggestive of causality in the analysis of pregnancy blood pressure and LBW (15).

Previous studies have mainly focused on the association between a single blood pressure measurement during pregnancy and LBW, while neglecting the dynamic changes in blood pressure patterns. Some studies suggest that stable blood pressure trajectories may have a protective effect on fetal growth, whereas persistently elevated or highly fluctuating blood pressure trajectories may increase the risk of adverse pregnancy outcomes (16, 17). However, how different blood pressure trajectory patterns affect the risk of LBW remains to be further investigated.

In addition, the relationship between maternal blood pressure trajectories and LBW may be influenced by various factors, including maternal age, BMI, education level, economic status, smoking, and alcohol consumption behaviors (18, 19). For instance, older pregnant women (>40 years) and those with a BMI > 28 may be more likely to exhibit abnormal blood pressure trajectories, thereby increasing the risk of LBW (8, 20). To more accurately assess the independent effect of blood pressure trajectories on LBW, this study employed DAG methodology for covariate selection, aiming to reduce the influence of potential confounding factors. The DAG method is widely used in causal inference studies, helping researchers identify and adjust for potential confounders, thereby optimizing statistical models and enhancing the robustness of causal inference (15).

Building on this background, this study utilized the NHANES 2005–2006 database and applied GBTM to identify maternal blood pressure trajectories during pregnancy and assess their relationship with LBW. Additionally, the study explored potential moderating effects of various population characteristics (such as age, BMI, and education level) on this relationship through stratified analyses, and employed DAG for suggestive of causality. The results of this study may provide new epidemiological evidence for managing maternal blood pressure during pregnancy and offer potential intervention strategies for preventing LBW.

## Methods

### Data sources and study population

This study utilized the NHANES 2005–2006 dataset, organized by the National Center for Health Statistics (NCHS) and the Centers for Disease Control and Prevention (CDC). The dataset employs a multi-stage, stratified, probability sampling method to collect health data from a nationally representative sample of the U.S. population (21). The data includes various health indicators, including health behaviors, physical examinations, and laboratory tests, providing key health metrics such as maternal blood pressure measurements and neonatal birth weight. The study population consisted of pregnant women who participated in the NHANES 2005–2006 survey, with the following inclusion criteria: complete data for three pregnancy-related blood pressure measurements (systolic blood pressure, SBP; diastolic blood pressure, diastolic blood pressure, DBP); pulse pressure, PP) and complete neonatal birth weight records. Exclusion criteria included participants with missing data, those with pregnancy complications (such as gestational hypertension or diabetes), and pregnant women with chronic diseases (such as chronic hypertension or cardiovascular diseases). All participants provided written informed consent prior to data collection. Due to the use of de-identified publicly available data, and the absence of personal identifiers, this study did not require approval from an institutional review board. All results were reported in accordance with the Strengthening the Reporting of Observational Studies in Epidemiology (STROBE) guidelines to ensure transparency and reliability.

### Data selection

**Figure 1:**
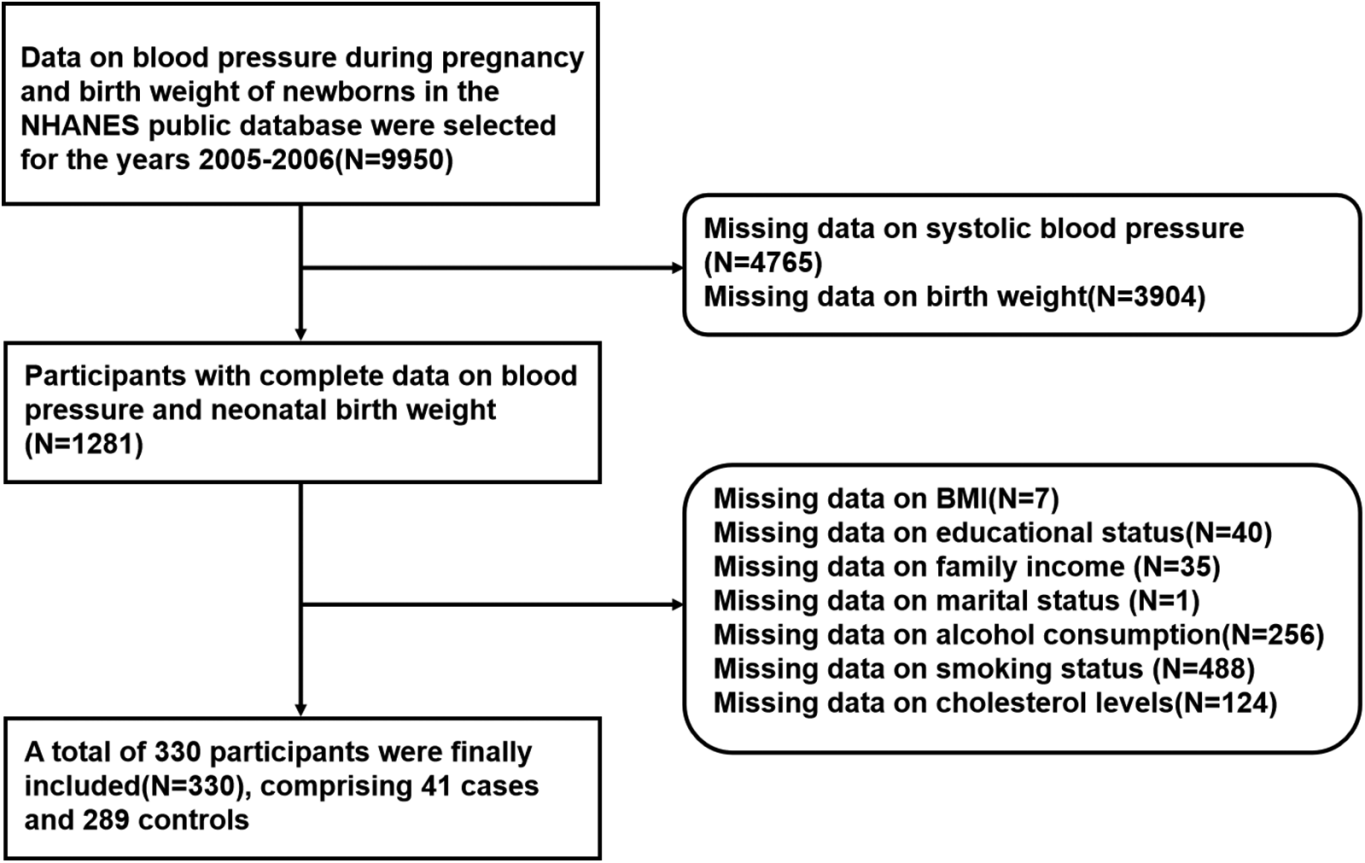
Flowchart of sample data selection.

To ensure data integrity and enhance the comparability of the results, the following data selection process was applied in this study (Figure 1). First, data on maternal blood pressure and neonatal birth weight from the NHANES 2005–2006 dataset were selected, with an initial sample size of 9950 participants. Next, participants with complete data on blood pressure and neonatal birth weight were selected, resulting in a total of 1,281 participants. Additionally, missing data were identified in variables such as BMI, education level, family income, marital status, alcohol consumption, smoking, and cholesterol levels, and participants with missing data in these variables were excluded. After the data cleaning process, a final sample of 330 participants with complete data was included, comprising 41 cases in the LBW group (birth weight <2,500 g) and 289 controls in the non-LBW group (birth weight ≥2,500 g).

### Study design

This study employed GBTM to analyze the trends in maternal blood pressure during pregnancy and to explore the association between different blood pressure trajectories and neonatal LBW. GBTM uses a finite mixture model to analyze longitudinal data and selects the optimal trajectory classification scheme based on the Bayesian Information Criterion (BIC) and the Likelihood Ratio Test (LRT). In this study, maternal blood pressure trajectories during pregnancy were categorized into high-medium, high-low, and medium-low patterns to reflect different trends in blood pressure changes over pregnancy.

The exposure variable in this study was maternal blood pressure trajectory, based on three measurements of SBP, DBP, and PP, taken at the 10th, 20th, and 30th weeks of pregnancy. The outcome variable was neonatal LBW, defined as birth weight <2,500 g. Additionally, demographic factors (age, race, education level, income, marital status), health behaviors (alcohol consumption, smoking during pregnancy), and biological factors (BMI, cholesterol levels) were included as covariates to control for potential confounding factors. To improve the scientific rigor of covariate selection, DAG analysis was used to screen variables, ensuring the rationality of model construction and minimizing confounding bias. The final selected covariates were used for subsequent statistical analyses.

## Statistical analysis

This study used GBT to analyze the relationship between maternal blood pressure trajectories and neonatal LBW, adjusting for covariates stepwise. The modeling was performed using R with the “traj” package. The Bayesian Information Criterion (BIC) was used to select the optimal number of trajectory groups. In this study, the number of groups was determined by minimizing the BIC, and the optimal solution was three trajectory groups based on maternal blood pressure measurements (systolic blood pressure, diastolic blood pressure, and pulse pressure). Three distinct blood pressure trajectory groups were initially identified during pregnancy using group-based trajectory modeling. In the multilevel model, individual-level random effects were included to account for the repeated blood pressure measurements within each participant. Specifically, the random effect in the multilevel model was applied to the participant level to account for the correlation between multiple blood pressure measurements collected from the same individual. Exposures were categorized into three types based on maternal blood pressure trajectories (systolic blood pressure, diastolic blood pressure, and pulse pressure). Inverse probability weighting (IPW) was calculated based on propensity scores derived from the covariates. The weights were stabilized to improve the precision of the potential causal relationship. Stabilized weights were used to reduce the potential bias in the estimation caused by extreme weights. Standardized mean differences were computed to compare the balance of measured variables between groups before and after applying IPW. In model 1, only blood pressure trajectories were adjusted to assess their primary impact on LBW. Model 2 incorporated maternal age to explore the moderating effect of age on the relationship between blood pressure trajectories and LBW. In model 3, additional covariates such as BMI, race, education level, income, marital status, alcohol consumption, smoking, and cholesterol levels were included to control for potential confounding factors. Finally, in model 4 (the final model), inverse probability weighting (IPW)-logistic regression was used based on model 3 to perform causal inference, reducing the impact of confounding factors and improving the precision of potential causal relationship.

To further investigate the relationship between blood pressure trajectories and LBW, this study performed stratified analyses to evaluate the impact of blood pressure trajectories on LBW across different subgroups. The stratification criteria included: age (≤40 years vs. >40 years), BMI (≤28 vs. >28), education level (high school or below vs. college and above), income (≤75,000 vs. >75,000), and lifestyle (alcohol consumption vs. no alcohol, smoking vs. no smoking). Stratified analyses help to examine whether different population characteristics influence the relationship between blood pressure trajectories and LBW. Additionally, IPW-Logistic regression was employed for potential causal relationship to simulate a randomized controlled trial (RCT) scenario, minimizing the impact of covariate imbalance. By calculating propensity scores based on covariates, weights were assigned to each individual, and samples were weighted to balance the covariate distribution across different blood pressure trajectory groups, thereby enhancing the accuracy of potential causal relationship. All statistical analyses were performed using R and the “traj” package for the group-based trajectory modeling. The Bayesian Information Criterion (BIC) was used to select the optimal number of trajectory groups.

## Results

### Comparison of demographic data between high-medium and medium-low groups

Table 1 summarizes the baseline demographic and clinical characteristics of participants in the high-medium and medium-low systolic pressure groups. No significant differences were observed between the two groups in terms of age (24.9 ± 10.8 vs. 24.3 ± 10.5 years, *P* = 0.826), BMI (28.8 ± 6.76 vs. 27.6 ± 6.59, *P* = 0.291), education level, income, marital status, smoking, or cholesterol status (*P* > 0.05 for all). However, a significant difference was observed in drinking behavior: 74.4% of participants in the high-medium group reported alcohol consumption, compared to 53.7% in the medium-low group (*P* < 0.001).

**Table 1:** Demographic data of high-medium and medium-low groups.

Subgroup	High-medium	Medium-low	*P* value
Age	24.9 ± 10.8	24.3 ± 10.5	0.826
BMI	28.8 ± 6.76	27.6 ± 6.59	0.291
Education			0.899
High school and below	148 (51.2%)	22 (53.7%)	
College and above	141 (48.8%)	19 (46.3%)	
Income			0.745
≤75,000	222 (76.8%)	33 (80.5%)	
>75,000	67 (23.2%)	8 (19.5%)	
Marital status			>0.999
Married	151 (52.2%)	21 (51.2%)	
Others	138 (47.8%)	20 (48.8%)	
Drinking			<0.001
No	74 (25.6%)	19 (46.3%)	
Yes	215 (74.4%)	22 (53.7%)	
Smoking			0.666
No	169 (58.5%)	26 (63.4%)	
Yes	120 (41.5%)	15 (36.6%)	
Cholesterol			0.166
No	171 (59.2%)	19 (46.3%)	
Yes	118(40.8%)	22(53.7%)	

### Consistency and stability analysis: reproducibility assessment of blood pressure measurements during pregnancy

**Figure 2:**
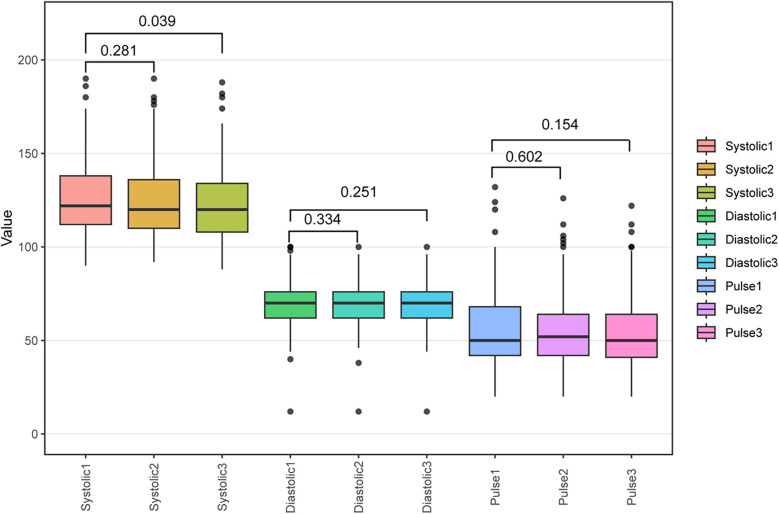
Box plot analysis of three blood pressure measurements during pregnancy.

To assess the stability of blood pressure measurements during pregnancy, this study compared three repeated measurements of SBP, DBP, and PP taken at the 10th, 20th, and 30th weeks of pregnancy. These measurements were collected using standardized automatic blood pressure measurement devices and visualized their variations using box plots (Figure 2). The results indicate that a significant difference was observed only between the first (Systolic1) and third (Systolic3) SBP measurements (*P* = 0.039), whereas no significant differences were found in other blood pressure measurements, including DBP and PP (*P* > 0.05). These findings suggest that SBP may fluctuate over time, particularly between early and later measurements, highlighting the need for careful monitoring of its dynamic changes during pregnancy. In contrast, DBP and PP remained stable across the three measurements, indicating good reproducibility and reliability as blood pressure assessment indicators during pregnancy.

### Maternal blood pressure trajectories and dynamic changes during pregnancy

**Figure 3:**
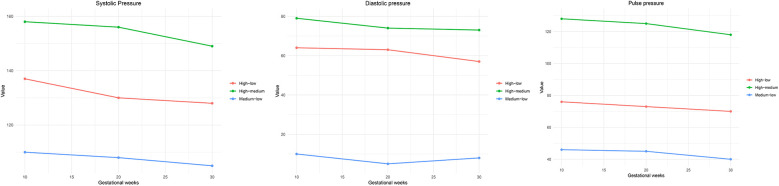
Maternal blood pressure trajectory model (GBTM) analysis results. This figure displays three distinct trajectory groups for **(A)** systolic blood pressure, **(B)** diastolic blood pressure, and **(C)** pulse pressure across gestational weeks 10, 20, and 30. The identified trajectories are labeled as high–low, high–medium, and medium–low, reflecting different trends in blood pressure changes over time. Pulse pressure was calculated as the difference between systolic and diastolic blood pressure.

GBTM identified three distinct maternal blood pressure patterns for each indicator throughout pregnancy (Figure 3). For systolic blood pressure (SBP), the high–medium group maintained the highest levels with a slight decline over time, the high–low group showed a moderate initial level with a gradual decrease, while the medium–low group presented the lowest SBP levels and a steady downward trend. DBP trajectories demonstrated relative stability in the high–medium group, a modest decline in late pregnancy in the high–low group, and a shallow U-shaped pattern in the medium–low group, characterized by a slight mid-pregnancy dip followed by a late rebound. In terms of PP, the high–medium group exhibited the highest values with a slight decrease, the high–low group remained intermediate and stable, and the medium–low group maintained the lowest PP with minimal fluctuation. These trajectory characteristics suggest that persistently elevated SBP and PP, especially in the high–medium group, may reflect increased vascular stiffness or subclinical hypertensive changes during pregnancy, which are known to impair placental perfusion and elevate the risk of adverse outcomes such as low birth weight (10, 14). In contrast, the declining SBP and stable, low DBP and PP observed in the medium–low group may indicate favorable cardiovascular adaptation. The mild rebound of DBP in late gestation seen in this group could represent a physiological response to increasing circulatory demands. Collectively, these findings suggeated that both the level and pattern of maternal blood pressure changes during pregnancy are critical for predicting perinatal outcomes.

### Impact of blood pressure trajectories on LBW

As shown in Table 2, this study analyzed the relationship between maternal blood pressure trajectories and LBW using multilevel logistic regression models. The analysis was conducted on 330 pregnant women from the NHANES 2005–2006 database. Model 1 adjusted for blood pressure trajectories only. The results indicated that the high-medium systolic pressure trajectory had an odds ratio (OR) of 3.520 [95% confidence interval (CI): 1.422–8.713], suggesting a significant association between maternal systolic pressure trajectories and LBW. Additionally, the high-medium diastolic pressure trajectory had an OR of 0.935 (95% CI: 0.486–1.792), showing some degree of association. However, the high-medium pulse pressure trajectory had an OR of 1.658 (95% CI: 0.086–10.396), indicating a weaker relationship.

**Table 2:** Association between maternal blood pressure trajectories and LBW across different pressure loci.

Group	Systolic pressure locus		Diastolic pressure locus		Pulse pressure locus	
	High-medium	Medium-low	High-medium	Medium-low	High-medium	Medium-low
	(*n* = 44)	(*n* = 169)	(*n* = 168)		(*n* = 7)	(*n* = 224)
OR (95% CI)						
Model 1	3.520 (1.422- 8.713)	2.164 (1.023–4.575)	0.935 (0.486–1.792)	-	1.658 (0.086–10.396)	2.580 (1.325–5.043)
Model 2	4.262 (1.576–11.523)	2.432 (1.104–5.355)	0.934 (0.486–1.792)	-	2.439 (0.121–17.124)	3.506 (1.579–8.022)
Model 3	7.833 (2.253–27.238)	3.243 (1.246–8.436)	0.784 (0.388–1.559)	-	1.831 (0.086–14.342)	3.874 (1.656–9.432)
Model 4	4.479 (2.541–7.895)	2.145 (1.247–3.690)	0.788 (0.491–1.265)	-	1.058 (0.374–2.995)	3.599 (2,098–6.175)

Note: Odds ratios (OR) and 95% confidence intervals (CI) were estimated using multilevel logistic regression models.

Model 1 was adjusted for maternal age, BMI, and race.

Model 2 was adjusted for maternal age, BMI, race, and education level.

Model 3 was adjusted for maternal age, BMI, race, education level, and income.

Model 4 was adjusted for maternal age, BMI, race, education level, income, alcohol consumption, smoking status, and cholesterol levels.

In Model 4, inverse probability weighting (IPW) was applied to minimize residual confounding.

The reference group for all comparisons was the group with the lowest blood pressure trajectory in each pressure locus.

The number of participants in the medium-low diastolic pressure group was too small for valid comparison and was excluded from the analysis.

SBP, systolic blood pressure; DBP, diastolic blood pressure; PP, pulse pressure.

In model 2, maternal age was added as a covariate to examine its moderating effect on the relationship between blood pressure trajectories and LBW. The results showed that maternal age played a moderating role. The OR for the high-medium systolic pressure trajectory increased to 4.262 (95% CI: 1.576–11.523), suggesting that age strengthens the relationship between systolic blood pressure trajectories and LBW. Other blood pressure trajectory groups showed similar trends, indicating that age plays a moderating role in the relationship between blood pressure trajectories and LBW.

In model 3, the relevant covariates are further added. The results showed that the OR for the high-medium systolic pressure trajectory was 7.833 (95% CI: 2.253–27.238), significantly increasing the risk of LBW. Moreover, OR values for other blood pressure trajectory groups also changed substantially, indicating that these covariates played a moderating role in the relationship between blood pressure trajectories and LBW.

Finally, in model 4, based on model 3, IPW logistic regression was used for potential causal relationship, further controlling for confounding factors. The results showed that the OR for the high-medium systolic pressure trajectory was 4.479 (95% CI: 2.541–7.895), further supporting the potential causal relationship between blood pressure trajectories and LBW. The analysis of other blood pressure trajectory groups also showed that the IPW method effectively reduced the impact of confounding factors, enhancing the reliability of association.

### Stratified analysis of systolic pressure locus and LBW

Due to the limited sample size for DBP and PP trajectories, stratified analysis was only conducted to examine the relationship between systolic pressure locus and LBW in 330 pregnant women from the NHANES 2005–2006 database, as shown in Table 3. The results indicate that maternal age, BMI, education level, income, marital status, alcohol consumption, and cholesterol levels may be important modifiers of this association.

**Table 3:** Stratified analysis of systolic pressure locus and LBW.

Subgroup	Systolic pressure locus					
	High-medium			Medium-low		
	OR (95% CI)	*P*	*P* (interaction)	OR (95% CI)	*P*	*P* (interaction)
Age			0.358			0.999
≤40	0.878 (0.876–8.80)	<0.001		0.829 (0.121–5.671)	0.848	
>40	5.475 (2.896–10.350)	<0.001		2.526 (1.360–4.693)	0.003	
BMI			0.875			0.053
≤28	2.429 (1.046–5.637)	0.039		0.575 (0.244–1.354)	0.205	
>28	6.801 (2.589–17.862)	<0.001		4.663 (1.950–11.150)		<0.001
Education		0.007				0.947
High school and below	11.960 (5.357–26.701)	<0.001		2.410 (1.096–5.292)	0.028	
College and above	0.841 (0.194–3.645)	0.817		2.096 (0.939–4.679)	0.071	
Income			0.050			0.050
≤75,000	3.849 (2.044–7.248)	<0.001		1.745 (0.948–3.212)	0.073	
>75,000	2.021 (0.293–13.92)	0.475		1.671 (0.402–6.955)	0.480	
Marital status			0.002			0.525
Married	3.920 (1.867–8.229)	<0.001		1.956 (0.958–3.994)	0.066	
Others	3.028 (1.179–7.772)	0.021		2.144 (0.907–5.067)	0.082	
Drinking			0.002			<0.001
No	18.992 (6.002–60.095)	<0.001		6.959 (2.551–18.984)	<0.001	
Yes	3.0129 (1.443–6.293)	0.003		1.385 (0.672–2.857)	0.377	
Smoking			0.780			0.987
No	5.875 (2.538–13.59)	<0.001		2.367 (1.135–4.937)	0.022	
Yes	6.600 (2.636–16.52)	<0.001		1.971 (0.777–5.003)	0.153	
Cholesterol			0.281			0.106
No	1.712 (0.769–3.812)	0.188		1.111 (0.553–2.235)	0.767	
Yes	14.388 (5.226–39.607)	<0.001		4.889 (1.950–12.258)	<0.001	

Note: The model adjusted for maternal age, BMI, education level, income, marital status, and drinking behavior. The analysis was based on model 4, IPW- logistic regression. The remaining covariates were adjusted.

In the age-stratified analysis, the association between high-medium SPL and LBW was more pronounced in mothers aged > 40 years (OR = 5.475, *P* < 0.001) compared to those aged ≤40 years (OR = 0.878, *P* < 0.001). The BMI-stratified analysis showed that the association was stronger in the BMI > 28 group (OR = 6.801, *P* < 0.001) than in the BMI ≤ 28 group (OR = 2.429, *P* = 0.039). Mothers with lower education levels (high school or below) exhibited the highest LBW risk in the high-medium SPL group (OR = 11.960, *P* < 0.001), whereas the association was not significant in those with higher education (*P* = 0.817). In the low-income group (≤75,000 USD), the association was stronger (OR = 3.849, *P* < 0.001), while no significant relationship was observed in the high-income group (*P* = 0.475). Regarding marital status, married women in the high-medium SPL group had a higher LBW risk (OR = 3.920, *P* < 0.001). For lifestyle factors, the association between SPL and LBW was more pronounced in non-drinkers (OR = 18.992, *P* < 0.001), whereas smoking status had a minimal effect on this relationship (*P* for interaction = 0.780). Additionally, in the abnormal cholesterol group, the association between high-medium SPL and LBW was stronger (OR = 14.388, *P* < 0.001), while no significant relationship was found in those with normal cholesterol levels (*P* = 0.188).

In summary, advanced maternal age (>40 years), higher BMI (>28), lower education levels, lower income, non-drinking status, and abnormal cholesterol levels may enhance the association between SPL and LBW. These findings highlight the importance of maternal demographic and lifestyle factors in pregnancy management.

### Association and covariate selection: DAG analysis

**Figure 4:**
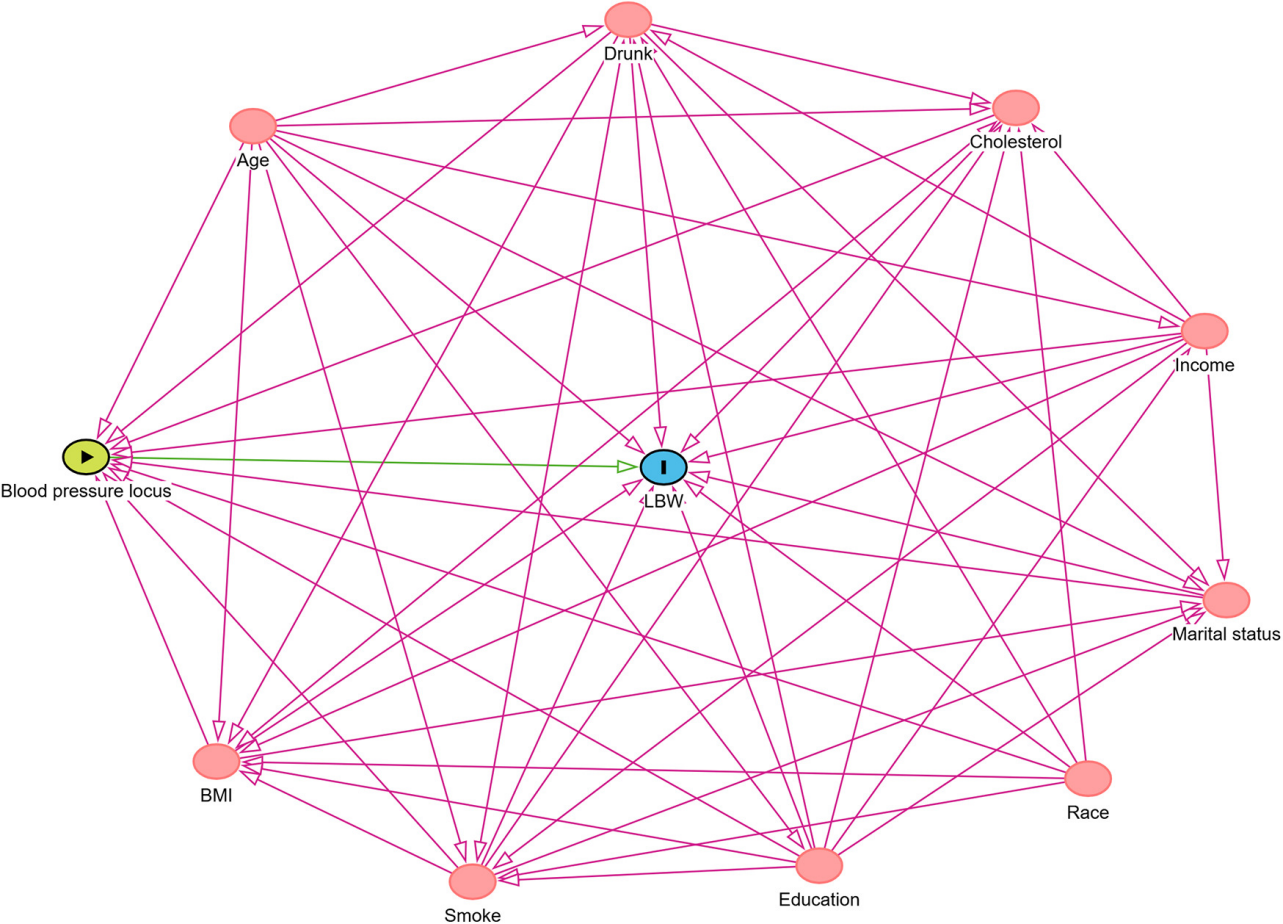
DAG analysis for covariate selection. The green node represents the exposure variable (blood pressure trajectory), the blue node represents the outcome variable (LBW), and the pink nodes represent potential confounders, including age, BMI, race, education level, income, marital status, alcohol consumption, smoking, and cholesterol levels.

To explore the potential association between blood pressure trajectories (exposure) and LBW (outcome), this study utilized DAG approach for covariate selection and identification of potential confounders among 330 pregnant women from the NHANES 2005–2006 database, as shown in Figure 4). The analysis involved adjusting for all confounders identified by the DAG to improve the robustness of the association estimation between blood pressure trajectories and LBW. The results showed that, after adjustment, blood pressure trajectories remained significantly associated with LBW, suggesting a potential independent effect and a possible causal relationship. Additionally, the DAG results indicated that, although variables such as age, BMI, education level, income, marital status, alcohol consumption, smoking, and cholesterol levels had some influence on LBW, they did not fully explain the effect of blood pressure trajectories.

## Discussion

This study, based on the NHANES 2005–2006 database, utilized GBTM to identify maternal blood pressure trajectories during pregnancy and examine their relationship with LBW. The results showed that maternal blood pressure trajectories during pregnancy exhibited distinct grouping characteristics. Mothers with a high-medium systolic pressure trajectory had a significantly increased risk of LBW in their newborns (OR = 4.479, 95% CI: 2.541–7.895, *P* < 0.001). Further stratified analyses revealed that this association was more pronounced among mothers aged >40 years, with a BMI > 28, low income, non-drinkers, and those with abnormal cholesterol levels. Additionally, DAG analysis further supported the independent effect of maternal blood pressure trajectories on LBW.

Maternal blood pressure trajectories may influence fetal growth and birth weight through various physiological mechanisms. Although women with diagnosed chronic hypertension and gestational diabetes were excluded from this study, it is possible that some participants—particularly those in the high-medium blood pressure trajectory group—may have exhibited subclinical vascular dysfunction or pre-hypertensive states. These latent abnormalities, although not clinically apparent, could still impair placental perfusion and fetal nutrient exchange, thereby contributing to the observed associations (22, 23). Prior studies have shown that hypertensive conditions are associated with inadequate placental blood flow, reduced oxygen and nutrient delivery to the fetus, and an increased risk of intrauterine growth restriction (IUGR) and LBW (24–26). Moreover, dynamic fluctuations in blood pressure may reflect maternal vascular maladaptation, such as decreased arterial compliance or increased vascular contractility, which could further compromise uteroplacental circulation (22, 27). This study found that even in the absence of a diagnosis of HDP, pregnant women with a high-medium systolic pressure trajectory still faced a higher risk of LBW, suggesting that it is not only the absolute blood pressure values but also the patterns of its changes over time that may have significant effects on fetal growth.

Stratified analysis results showed that the relationship between maternal blood pressure trajectories and low birth weight (LBW) was more pronounced in older mothers (>40 years) and those with a BMI > 28. Older pregnant women may exhibit early vascular aging or subclinical arterial stiffness, which—although not clinically diagnosed as hypertension—can impair vascular adaptability during pregnancy and potentially reduce placental perfusion (23, 28). Similarly, pregnant women with elevated BMI may present with insulin resistance and low-grade systemic inflammation, which are not limited to overt gestational diabetes but may reflect underlying metabolic disturbances associated with vascular dysfunction (29, 30). These early or subclinical changes may act synergistically to exacerbate the adverse effects of abnormal blood pressure trajectories on fetal development, particularly in older or high-BMI pregnant women. Furthermore, this study found that the relationship between blood pressure trajectories and LBW was more significant in pregnant women with low income and abnormal cholesterol levels. The impact of socioeconomic status on maternal health has been well-documented in numerous studies (31). Low-income pregnant women may face higher risks of malnutrition, increased pregnancy stress, and limited access to medical resources, all of which can further exacerbate the adverse effects of blood pressure on fetal growth by affecting placental function (32). On the other hand, abnormal cholesterol levels may be associated with vascular dysfunction and metabolic abnormalities, which could influence blood pressure regulation during pregnancy and increase the risk of LBW (33). DAG analysis results showed that even after adjusting for potential confounders such as age, BMI, education level, income, marital status, alcohol consumption, smoking, and cholesterol levels, blood pressure trajectories still had a significant impact on LBW. These findings suggest that maternal blood pressure trajectories may be an independent predictor of LBW, rather than merely an indirect reflection of other confounding factors.

The covariate selection based on DAG further support for a potential causal association between blood pressure trajectories and LBW, highlighting the clinical importance of monitoring blood pressure patterns throughout pregnancy.

This study has several limitations. First, the NHANES database includes only three blood pressure measurements during pregnancy, which may not fully reflect the long-term trends in maternal blood pressure changes over the course of pregnancy. Future studies could integrate higher-frequency blood pressure monitoring data to derive refined blood pressure trajectory patterns. Second, this study did not include maternal lifestyle factors (such as diet and physical activity), which may influence maternal blood pressure trajectories and their impact on LBW (34, 35), we further acknowledge that additional important confounding variables—including maternal dietary intake, physical activity levels, medication use (especially antihypertensive drugs), and pregnancy-related complications (e.g., gestational diabetes, preeclampsia)—were not systematically available or complete in the selected sample of pregnant women from NHANES. Therefore, these variables could not be included in the current analysis, which may result in residual confounding and bias the reported associations. Future research should incorporate such covariates to better isolate the independent effect of blood pressure trajectories on LBW. Furthermore, since the NHANES data are observational, although a potential causal relationship was explored using DAG in this study, RCTs are needed to further validate the suggestive causal link between blood pressure trajectories and LBW. Despite statistical adjustments using DAG and IPW, the potential for residual confounding in observational studies remains. Therefore, these findings should be interpreted as suggestive of causality rather than definitive evidence. Additionally, it is important to note that the data used in this study were derived from the American population, which may limit the generalizability of the findings to other populations. Lastly, due to the sampling methodology used in NHANES, the sample size in this study was relatively small. Future studies should validate these findings using larger datasets to enhance the robustness and generalizability of the conclusions.

## Conclusion

This study found that mothers with a high-medium systolic blood pressure trajectory during pregnancy had a significantly increased risk of LBW in their newborns, with this association being more pronounced in older mothers, those with a BMI >28, low income, and those with abnormal cholesterol levels. DAG analysis further suggested a potential causal association between maternal blood pressure trajectories based on three time points and LBW. This study emphasizes the importance of monitoring blood pressure trajectories during pregnancy and suggests that early identification of high-risk pregnant women and personalized interventions may help reduce the risk of LBW.

## Data Availability

The original contributions presented in the study are included in the article/Supplementary Material, further inquiries can be directed to the corresponding author.
